# Predicting radiotherapy efficacy and prognosis in tongue squamous cell carcinoma through an in-depth analysis of a radiosensitivity gene signature

**DOI:** 10.3389/fonc.2024.1334747

**Published:** 2024-08-26

**Authors:** Jinzhi Lai, Hainan Yang, Junjun Chen, Shoubo Chen, Xiaofang Chen

**Affiliations:** ^1^ Department of Oncology, The Second Affiliated Hospital of Fujian Medical University, Quanzhou, Fujian, China; ^2^ Department of Ultrasound, First Affiliated Hospital of Xiamen University, Xiamen, Fujian, China; ^3^ National Health Commission (NHC) Key Laboratory of Personalized Diagnosis and Treatment of Nasopharyngeal Carcinoma, Jiangxi Cancer Hospital of Nanchang Medical College, Nanchang, Jiangxi, China; ^4^ Department of Orthopaedics, The Second Affiliated Hospital of Fujian Medical University, Quanzhou, Fujian, China; ^5^ Department of Otolaryngology, The Second Affiliated Hospital of Fujian Medical University, Quanzhou, Fujian, China

**Keywords:** tongue squamous cell carcinoma, radiosensitivity, radiotherapy, prognosis, prognostic index

## Abstract

**Background:**

Tongue squamous cell carcinoma (TSCC) is a prevalent tumor that affects many people worldwide. Radiotherapy is a common treatment option, but its efficacy varies greatly. This study seeks to validate the identified gene signature associated with radiosensitivity in TSCC, and its potential in predicting radiotherapy response and prognosis.

**Methods:**

We analyzed 122 TSCC patients from TCGA database using the radiosensitivity signature and classified them into radiosensitive (RS) and radioresistant (RR) groups. Immune infiltration analysis methods were applied to investigate the immune status between different subgroups. Immunophenotype Score (IPS) and pRRophetic algorithm were employed to estimate the efficiency of treatment. A radioresistant TSCC cell line was established by gradually increasing radiation doses. Cell radiosensitivity was evaluated using the CCK-8 and colony formation assays. The expression of radiosensitivity-related genes was validated by qRT-PCR.

**Results:**

Our study validated the predictive capacity of a previously identified “31-gene signature” in the TCGA-TSCC cohort, which effectively stratified patients into RS and RR groups. We observed that the RS group exhibited superior overall survival and progression-free survival rates relative to the RR group when treated with radiotherapy. The RS group was significantly enriched in most immune-related hallmark pathways, and may therefore benefit from immune checkpoint inhibitors. However, the RS group displayed lower sensitivity to first-line chemotherapy. A radioresistant TSCC cell line (CAL-27R) exhibited increased clonogenic potential and cell viability following irradiation, accompanied by downregulation of three radiosensitivity-related genes compared to its parental non-resistant cell (CAL-27). In addition, we constructed and validated a radiosensitivity-related prognostic index (PI) using 4 radiosensitivity-related genes associated with TSCC prognosis.

**Conclusion:**

We assessed the ability of the radiosensitivity gene signature to predict outcomes in TSCC patients. our research provided valuable insights into the molecular pathways associated with radiosensitivity in TSCC and offered clinicians a practical tool to predict patient radiotherapy effectiveness and prognosis.

## 1 Introduction

Tongue squamous cell carcinoma (TSCC) is a highly prevalent malignant tumor of head and neck region. It is known for its more aggressive behavior compared to conventional squamous cell carcinoma (1). Despite the progress made in cancer diagnosis and therapeutics over the past few decades, the recurrence of TSCC is common and the prognosis remains unsatisfactory (2). Surgery and perioperative radiotherapy are the most common treatment options for TSCC. For most of TSCC patients, radiotherapy is a highly effective modality for treating TSCC of different stages, and it plays a critical role in reducing mortality rate of TSCC patients (3). Although significant advancements in radiotherapy, radioresistance remains a significant challenge that restricts the clinical efficacy of radiotherapy. Tumor radioresistance is an important risk factor for developing locoregional relapse and distant metastasis, especially for TSCC patients (4). Unfortunately, there are no efficient radiosensitivity biomarkers that can help select TSCC patients who are more likely to respond effectively to radiotherapy. Further research is still necessary to improve radiotherapy efficacy and establish new biomarkers for better selection of TSCC patients.

It is widely acknowledged the genomic feature could be a potential factor contributing to the heterogeneity of radiotherapy efficiency (5). In recent years, there has been much attention given to identifying radiation-specific biomarkers at the genome level to improve the effectiveness of radiotherapy (6). Constructing a gene signature is a reliable and practical way to screen for radiosensitive patients and support clinical decision-making. Several gene signatures have been developed to predict radiosensitivity in diverse cancer types, including head and neck squamous cell carcinoma (HNSCC) (7), breast cancer, and lung cancer (8). In addition, several teams have focused on constructing pan-cancer radiation-specific models that could transform the medical practice of radiation oncology (9, 10). Therefore, identifying radiosensitivity genes as biomarkers holds great potential for optimizing radiotherapy strategies.

With the advent and commercialization of high-throughput technology, gene sequencing has become an important tool in developing radiosensitivity prediction models and understanding the mechanism of radiosensitivity in various types of cancer. A notable example is the radiosensitivity signature, a set of 31 genes derived from the radiosensitivity profiling of the NCI-60 cell line panel (11). This signature can stratify patients into radioresistant (RR) and radiosensitive (RS) groups, which have varying clinical outcomes and effectiveness of radiotherapy. The signature has been validated in multiple types of tumors, including breast cancer, lower grade glioma, and pancreatic cancer. Additionally, combined immunotherapy and radiotherapy, specifically utilizing immune checkpoint blockade, may be beneficial for a subset of patients with high PD-L1 expression within the radioresistant group, particularly in breast cancer and low-grade glioma (12–14). However, the 31-gene signature has not been validated for TSCC patients.

In our study, we aimed to validate the 31-gene signature associated with radiosensitivity in TSCC patients using the TCGA database. Patients were categorized into RS and RR groups using the 31-gene signature and observed significant survival differences when TSCC patients received radiotherapy. To gain a deeper understanding of radiosensitivity in TSCC, we investigated the differences in functional enrichment, immune infiltration status, and response to different treatment strategies between the two groups. Additionally, we constructed a prognostic index to predict the prognosis of TSCC patients. Our research demonstrates the potential predictive value of the 31-gene radiosensitivity signature and may pave the way for more personalized approaches to cancer therapy in the future.

## 2 Materials and methods

### 2.1 Public data acquisition and the unsupervised clustering

The TCGA-TSCC dataset was sourced from the UCSC Xena database (https://xena.ucsc.edu/). The TCGA (The Cancer Genome Atlas) is a comprehensive cancer research program that provides a vast repository of genomic, clinical, and pathological data from over 33,000 cancer patients across 33 different cancer types. This comprehensive dataset has been a valuable resource for advancing cancer research, providing insights into the molecular mechanisms driving different cancer types. For this study, 122 TSCC patients were selected for further analysis based on the availability of both RNA-seq data and clinical information. Somatic variant mutation annotation format (MAF) was obtained using the “maftools” R package. The prognostic validation cohort, GSE41613 (n=97), comprised TSCC microarray data and associated clinical information obtained from the Gene Expression Omnibus (GEO) database (15).

The study leveraged a 31-gene signature, previously identified through a meta-analysis of four NCI-60 cancer cell line microarrays, for patient stratification based on predicted radiosensitivity (11, 16). This signature was selected due to its established association with radiosensitivity and its potential as a biomarker for predicting response to radiation therapy. The classification of patients into two groups or “clusters” was based on the 31-gene signature and was accomplished using a consensus clustering algorithm. Subsequently, the study examined whether survival outcomes following radiotherapy differed between the two clusters. The clusters were designated as the RS group or the RR group based on their respective prognoses following primary radiotherapy. Principal Component Analysis (PCA) and University Mobility in Asia and the Pacific (UMAP) analyses were utilized to assess the capability of the 31-gene signature to distinguish samples. The “ggplot2” and “umap” R packages were utilized for PCA and UMAP, respectively. Differentially expressed genes (DEGs) between the RS and RR groups were analyzed based on the cutoff criteria of |log2-fold change (FC)| > 1.5 and an adjusted p-value < 0.05.

### 2.2 Estimation of infiltration of immune cells and immune status

In this study, we conducted an analysis of tumor immune microenvironment using several computational tools. Firstly, We employed the “ESTIMATE” algorithm to assess immune score, stromal score, and tumor purity in each tumor sample, using gene expression profiles as input (17). This approach allowed us to determine the extent of immune cell infiltration and the overall purity of the sample. Then, we performed single-sample Gene Set Enrichment Analysis (ssGSEA) to assess the enrichment levels of 29 immunity-related signatures, encompassing various immune pathways and functions (18). TSCC patients were classified into high-immunity and low-immunity groups using unsupervised hierarchical clustering based on the ssGSEA scores of 29 immune signatures. Furthermore, we utilized the CIBERSORT computational tool to estimate the relative abundances of 22 types of tumor-infiltrating immune cells in each sample based on gene expression data (19). Results with a p-value ≤ 0.05 were considered eligible for further study. This approach allowed for a comprehensive evaluation of the immune status of each tumor sample and provided insights into the potential role of immune cell infiltration in tumor progression and response to treatment.

### 2.3 Functional enrichment and gene set variation analysis

GSVA was employed to evaluate the variation of pathway activity across different clusters in an unsupervised manner using the GSVA package. The gene set, ‘c2.cp.kegg.v7.5.1.symbols.gmt’, was retrieved from the Molecular Signatures Database (MSigDB) and selected as the background gene set for this analysis. Differential analysis of the Kyoto Encyclopedia of Genes and Genomes (KEGG) pathway between two clusters was performed using the “limma” R package (20, 21). Patients were clustered based on their pathway enrichment scores, with statistical significance determined by an adjusted p-value below 0.05.

### 2.4 Predicting immunotherapy response and assessing drug sensitivity

The Immunophenotype Score (IPS) is a comprehensive measure that quantifies the immunogenicity of solid tumors, which serves as an indicator of the potential response to immunotherapy. The IPS score ranges from 0 to 10 and is determined by the expression of specific gene sets. The Cancer Immunome Atlas (TCIA) database uses machine learning to develop a scoring system that quantifies the IPS (22). The efficacy of immunotherapy using anti-CTLA-4 and anti-PD-1 antibodies was predicted using the IPS for these blockers. To predict patient responses to chemotherapy and targeted therapies, we employed the “pRRophetic” R package (23). This package leverages data from the Genomics of Drug Sensitivity in Cancer (GDSC) database, which contains information on the responses of various cancer cell lines to chemotherapeutic and targeted agents. The pRRophetic package utilizes this data to predict the sensitivity of a specific cancer cell line to a particular drug (24). The IC50 value represents the concentration of a drug required to inhibit 50% of cell growth, with lower IC50 values indicating greater sensitivity of tumor cells to specific chemotherapeutic and targeted agents.

### 2.5 Construction of the radiosensitivity-related prognostic index

Our study employed a univariate Cox regression analysis in the training set to screen for radiosensitivity-related prognostic genes. Statistical significance was established at a p-value threshold of 0.05, and genes meeting this criterion were selected for further analysis. We employed least absolute shrinkage and selection operator (LASSO)-penalized Cox regression analysis to identify the most reliable predictors and construct a prognostic model. This approach allowed us to determine the most important genes for prognostic purposes. The final genes were selected, and a PI was generated using the formula: prognostic index (PI) = coef 1 × expgene1 + coef 2 × expgene2……coef n × expgene n. Patients with TSCC were divided into high-PI and low-PI groups based on the median PI, and survival was analyzed using the Kaplan-Meier method. The predictive value of the nomogram was verified using decision curve analysis (DCA) in comparison to other independent factors (25). The performance of the PIs was evaluated using a time-dependent receiver operating characteristic (ROC) curve analysis. The ROC curves for the four previously reported models were obtained based on the TCGA-TSCC dataset. The riskscore was calculated for each TCGA-TSCC sample using the corresponding genes in the four models. The samples were then stratified into high-PI and low-PI groups based on the riskScores. Subsequently, time-dependent ROC curves were plotted, and the area under the curve (AUC) for 1-year, 3-year, and 5-year overall survival (OS) in the TCGA-TSCC datasets was calculated to evaluate the prognostic accuracy of the four models.

### 2.6 Cell culture and establishment of radioresistant TSCC cells

The Human TSCC cell line, CAL-27, was procured from the China Center for Type Culture Collection (CCTCC, Wuhan, China). The CAL-27 cell line was cultured in RPMI medium 1,640 (Corning, United States) supplemented with 10% fetal bovine serum (Corning, United States) and 1% antibiotics (Gibco-BRL, Gaithersburg, MD, United States) and incubated at a temperature of 37°C with saturated humidity and 5% CO2. Prior to use, CAL-27 cells were screened for mycoplasma contamination. Radioresistant CAL-27R was established by subjecting CAL-27 cells to repeated radiation exposure. Briefly, CAL-27 cells received a total radiation dose of 50 Gy, administered in seven fractions with increasing doses: 2 Gy, 4 Gy, 6 Gy, 8 Gy, and then three consecutive doses of 10 Gy (26). The radio-resistance of CAL-27R was assessed through cell proliferation assays.

### 2.7 Cell proliferation assays

The assessment of cell viability was conducted utilizing the CCK-8 assay in adherence to the manufacturer’s guidelines. The cells were seeded and placed in 96-well plates, followed by incubation. After 24 hours of incubation, the cells were exposed to 4 or 8 Gy irradiation. CAL-27 and CAL-27R cells were exposed to radiation for a total of 48 hours. Following the radiation, the optical density at 450 nm was measured using a microplate reader after adding 10 μl of Cell Counting Kit-8 solution (Dojindo, Kumamoto, Japan) to the cells and incubating them for three hours.

### 2.8 Colony formation assay

To assess clonogenic survival, both cell lines were seeded at a density of 500 cells per well in six-well plates and incubated overnight to allow for cell attachment. The following day, cells were irradiated with increasing doses of radiation (5 and 10 Gy). Cultures were maintained for 14 days, at which point colonies were visible. After fixation with 4% paraformaldehyde for 30 minutes, colonies were stained with 1% (w/v) crystal violet for 30 minutes, with the dye solution being reused. Colony efficiency was calculated as the number of colonies divided by the initial number of seeded cells, multiplied by 100%.

### 2.9 Quantitative real-time polymerase chain reaction

The RNA extraction process involved the use of Trizol reagent, following the protocol provided by the manufacturer (Invitrogen, San Diego, CA, USA). The concentration of the extracted RNA was determined using a NanoDrop 2000 spectrophotometer (ThermoFisher, USA). The cDNA synthesis was performed using the Transcriptor First Strand cDNA Synthesis Kit (Roche, Germany) on the extracted RNA. The SYBR Prime Script RT-PCR Kit (Invitrogen, USA) was utilized for quantitative real-time PCR (RT-qPCR), with primer sequences listed in **Supplementary Table S1**. The 2^-ΔΔ^CT method was used to calculate relative expression levels, with all results presented as fold changes relative to the internal control genes. Ct values were normalized to the geometric mean of GAPDH, an internal control gene, and all experiments were conducted in triplicate. The data obtained from this study were derived from three independent experiments, ensuring accuracy and reliability.

### 2.10 Statistical analysis

The statistical methods used for data calculation and comparison were incorporated into the analysis using R software version 4.1.3. To compare normally distributed data between two groups, a Student’s t-test was used. For categorical and pairwise features across different groups, the Chi-square test was employed. The Mann-Whitney U test was used to analyze statistically significant differences between two groups, and the Kruskal-Wallis test was utilized to evaluate statistically significant differences among multiple independent groups. When performing multiple dependent or independent statistical tests, a Bonferroni correction was applied to adjust for multiple comparisons. Pearson’s correlation test was used to assess correlations between normally distributed variables, and Spearman’s correlation test was used to evaluate correlations between variables that were not normally distributed. The Kaplan-Meier method and log-rank test were applied to analyze survival differences between two or more groups. All tests were two-sided, and statistical significance was defined as a p-value less than 0.05, unless otherwise specified.

## 3 Results

### 3.1 Validation of radiosensitivity gene signature in TSCC

**Figure 1:**
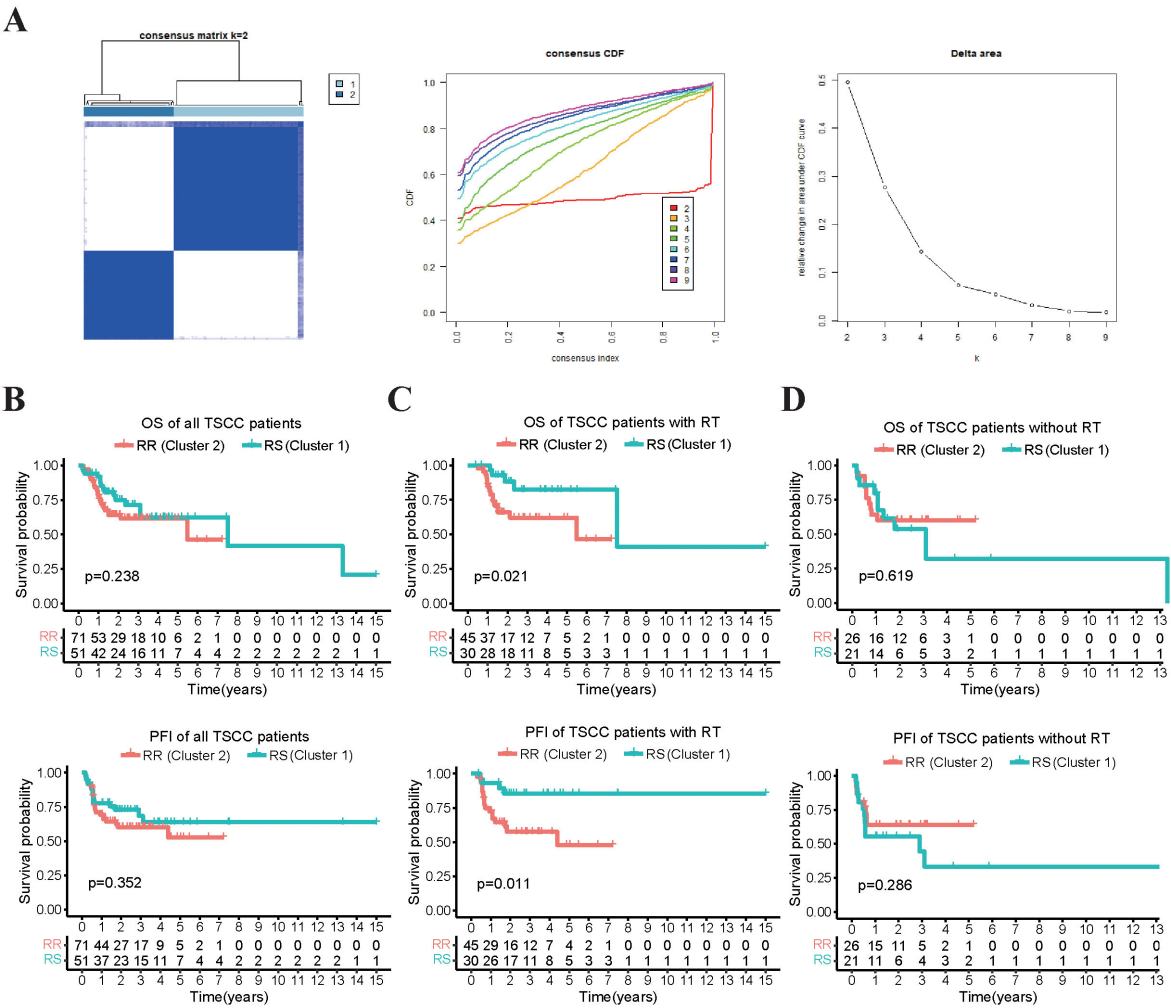
Survival analysis that compares the OS and PFI between two clusters of patients receiving radiotherapy and not. **(A)** The TSCC patients were subjected to consensus clustering based on 31 radiosensitivity-related genes, and the optimal number of clusters was verified using cumulative distribution function (CDF) curves. **(B)** The Kaplan-Meier plot illustrated that there was no significant difference in OS and PFI between the two clusters in all the patients. **(C)** patients in cluster 1 (RS group) who received radiotherapy had better OS and PFI compared to those in cluster 2 (RR group). **(D)** there was no significant difference in OS and PFI between the two clusters in the patients who did not receive radiotherapy.

We first validated the radiosensitivity predictive capacity of the 31-gene signature in TCGA-TSCC cohort. Using the consensus clustering algorithm, we divided TSCC patients into two clusters: cluster 1 (n = 51, 41.8%) and cluster 2 (n = 71, 58.2%), based on the gene expression levels of the 31-gene signature (**Figure 1A**). These clusters represented two different types based on the 31-gene signature. To validate these two clusters and identify the RS and RR groups, we performed survival analysis. The Kaplan-Meier curve showed no significant difference in OS and progression-free interval (PFI) between the cluster 1 and cluster 2 groups (**Figure 1B**). However, patients who received radiotherapy exhibited better OS and PFI in cluster 1 than cluster 2 (**Figure 1C**). No survival differences were observed in patients without radiotherapy between the two clusters (**Figure 1D**). Therefore, the 31-gene could potentially serve as a predictive biomarker for predicting the prognosis of TSCC patients after radiotherapy. We defined cluster 1 as the RS group and cluster 2 as the RR group. The RS group may benefit more from radiotherapy than the RR group.

### 3.2 The correlation between radiosensitivity group and tumor characteristics

**Figure 2:**
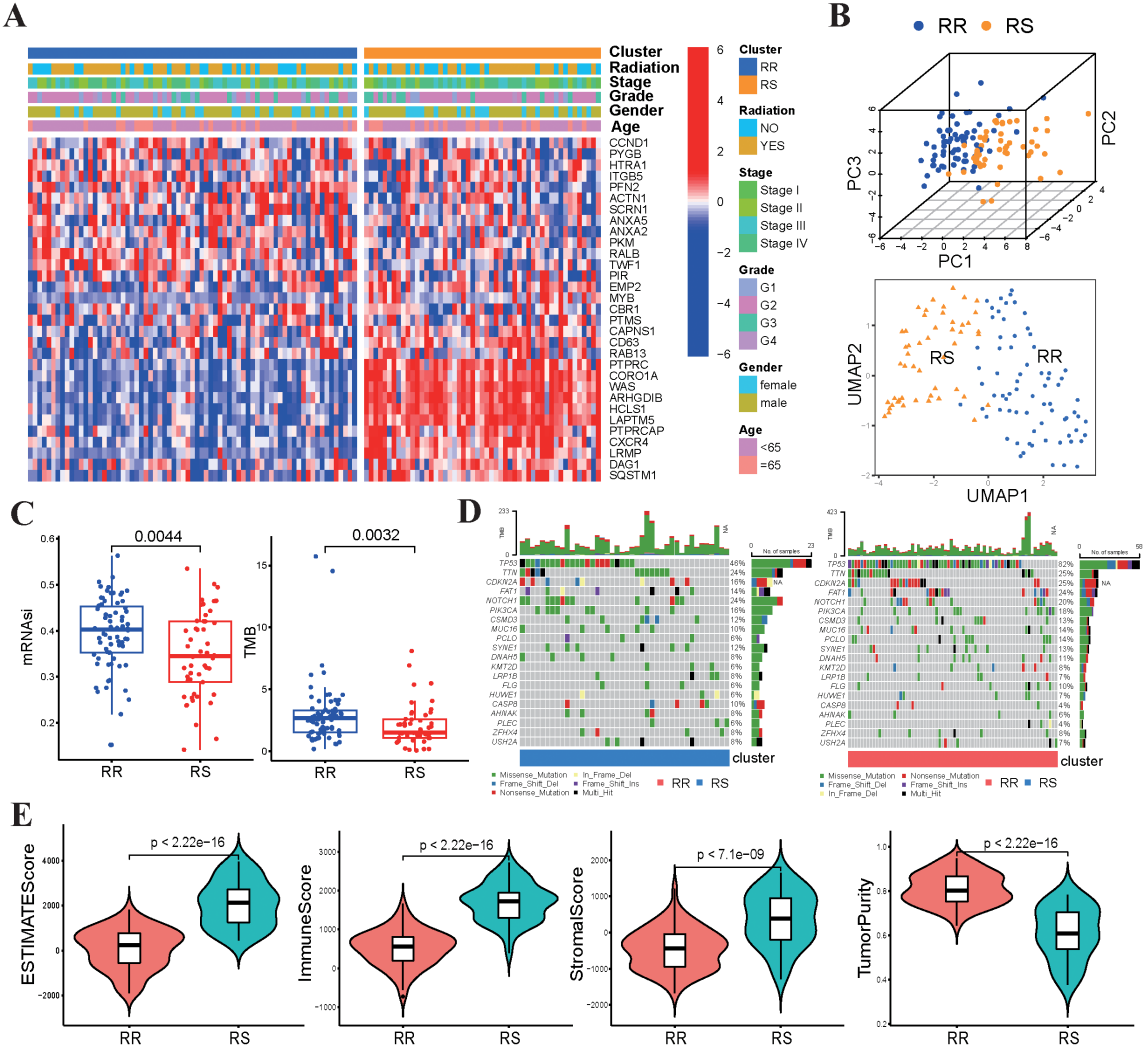
Relationship between radiosensitivity group and tumor characteristics. **(A)** The heatmap displayed the correlation between the expression levels of 31 genes and the clinical-pathological features of TSCC patients. **(B)** The PCA and UMAP plots are based on radiosensitivity gene signature of 31 genes in TSCC patients. **(C)** Comparisons of mRNAsi scores and TMB values between the RS group and RR group. **(D)** The waterfall plots depicted the most frequently mutated genes in the RS and RR groups, respectively. **(E)** Comparisons of the infiltration levels of estimate, stromal, immune scores and tumor purity in between two groups using boxplots.

The clinicopathological characteristics of patients were presented in **Table 1**. No statistically significant differences were found in clinical characteristics between the RS and RR groups (**Figure 2A**). The radiosensitivity signature was validated using PCA and UMAP, which classified TSCC patients into two clusters (**Figure 2B**). We subsequently compared the tumor characteristics between the RS group and the RR group. Tumor stemness, which represents the capacity for self-renewal and differentiation, has been shown to affect treatment resistance. We found that the stemness index (mRNAsi) and TMB were significantly lower in the RS group compared to the RR group (**Figure 2C**). Waterfall plots showed significant differences in the frequency of mutations across various genes between the two groups. The mutation frequency of TP53 was higher in the RR group compared to the RS group. On the other hand, the RS group was characterized by high-frequency mutations of NOTCH1, while the RR group had high mutation frequency of CDKN2A and FAT1 (**Figure 2D**). Furthermore, the ESTIMATE algorithm demonstrated that the estimate score, stromal score and immune scores of RS group were increased compared to those of the RR group, while tumor purity was decreased (**Figure 2E**). These results indicated that genomic differences between these two groups might influence tumor immune infiltration status and immunotherapy responsiveness.

**Table 1:** Relationship between radiosensitivity group and clinicopathological characteristics in TCGA-TSCC cohort.

Type	Total	RR group	RS group	P-value
Age				
<=65	91 (74.59%)	56 (78.87%)	35 (68.63%)	0.284
>65	31 (25.41%)	15 (21.13%)	16 (31.37%)	
Gender				
female	38 (31.15%)	21 (29.58%)	17 (33.33%)	0.8075
male	84 (68.85%)	50 (70.42%)	34 (66.67%)	
Grade				
G1	19 (15.57%)	14 (19.72%)	5 (9.8%)	0.2581
G2	76 (62.3%)	44 (61.97%)	32 (62.75%)	
G3	23 (18.85%)	12 (16.9%)	11 (21.57%)	
G4	4 (3.28%)	1 (1.41%)	3 (5.88%)	
Stage				
Stage I	9 (7.38%)	4 (5.63%)	5 (9.8%)	0.1636
Stage II	31 (25.41%)	20 (28.17%)	11 (21.57%)	
Stage III	36 (29.51%)	25 (35.21%)	11 (21.57%)	
Stage IV	46 (37.7%)	22 (30.99%)	24 (47.06%)	
M_stage				
M0	119 (97.54%)	70 (98.59%)	49 (96.08%)	0.7707
M1	3 (2.46%)	1 (1.41%)	2 (3.92%)	
N_stage				
N0	62 (50.82%)	41 (57.75%)	21 (41.18%)	0.0785
N1	25 (20.49%)	16 (22.54%)	9 (17.65%)	
N2	33 (27.05%)	13 (18.31%)	20 (39.22%)	
N3	2 (1.64%)	1 (1.41%)	1 (1.96%)	
T_stage				
T1	13 (10.66%)	6 (8.45%)	7 (13.73%)	0.736
T2	45 (36.89%)	28 (39.44%)	17 (33.33%)	
T3	48 (39.34%)	27 (38.03%)	21 (41.18%)	
T4	16 (13.11%)	10 (14.08%)	6 (11.76%)	
Radiation				
NO	47 (38.52%)	26 (36.62%)	21 (41.18%)	0.7478
YES	75 (61.48%)	45 (63.38%)	30 (58.82%)	

### 3.3 Functional annotations and tumor immune infiltration status of TSCC associated with the radiosensitivity

**Figure 3:**
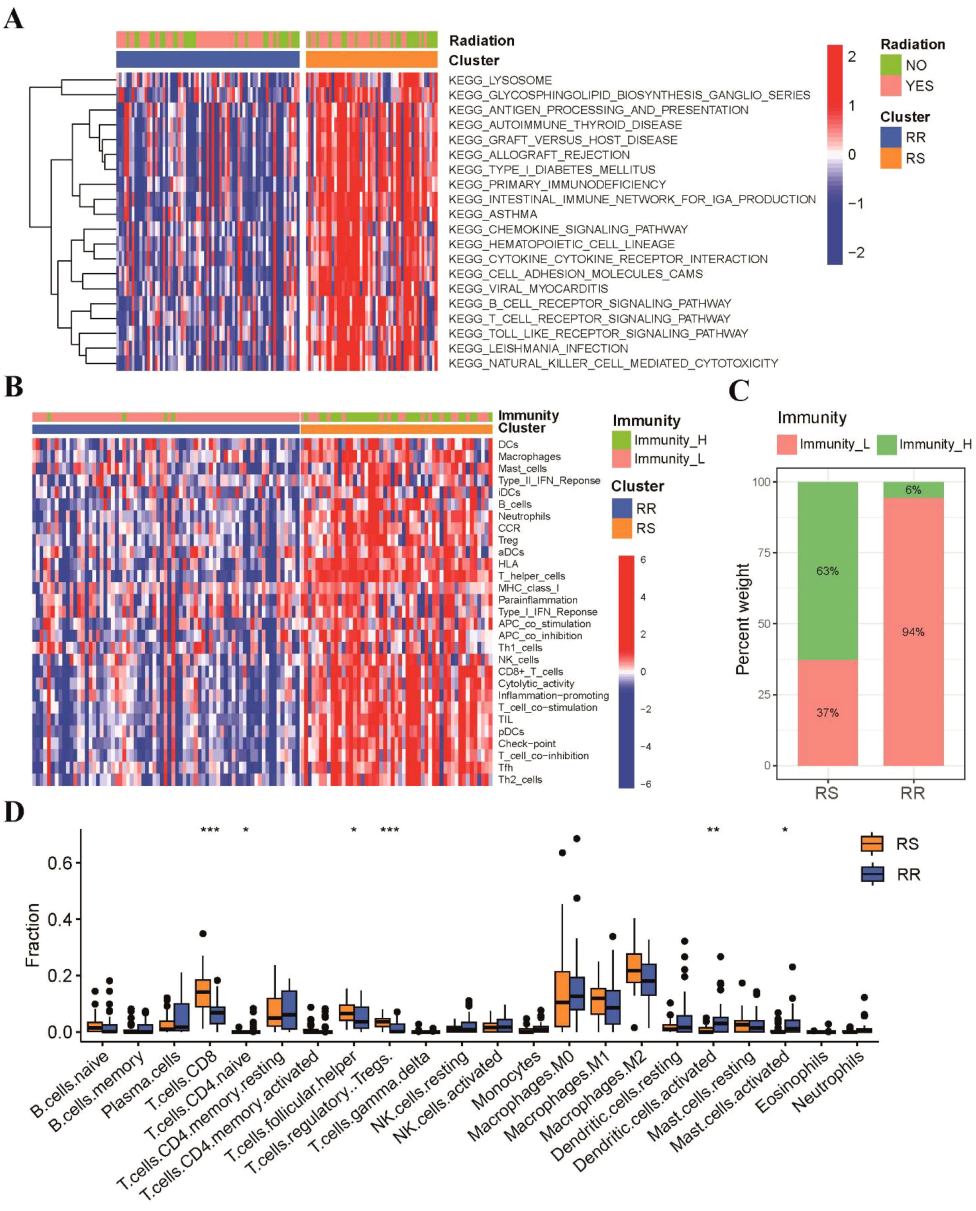
These two groups possessed different functional annotations and tumor immune infiltration status. **(A)** The heatmap of GSVA result showed the top 20 KEGG enriched pathways between RS and RR groups. **(B)** The immune subgroups of TSCC patients were categorized based on the enrichment of 29 immune hallmarks. **(C)** A stacked histogram illustrated the proportions of high-immunity and low-immunity patients in the two groups. **(D)** Comparisons of the proportions of 22 immune-infiltrating cells between the two groups. * p<0.05, ** p<0.01, *** p<0.001.

To investigate the molecular pathways associated with radiosensitivity in TSCC, we used GSVA enrichment analysis to compare KEGG pathway activity between the RS and RR groups. Our findings revealed that pathways linked to immune responses, such as antigen processing and presentation, T-cell receptor signaling pathway and natural killer cell mediated cytotoxicity pathway, were enriched in RS group (**Figure 3A**). Subsequently, ssGSEA algorithms was used to assess tumor immune infiltration based on the enrichment of 29 immune hallmarks. Our results indicated that the RS group was significantly enriched in most of immune-related hallmark pathways (**Figure 3B**). We clustered TSCC patients into high-immunity and low-immunity subgroups and found that the RS group consisted mostly of high-immunity patients (**Figure 3C**). Then, CIBERSORT was employed to quantify the abundances of 22 immune-infiltrating cells between the two groups. Our results demonstrated that CD8 T cells, regulatory T cells and T follicular helper (Tfh) cells were significantly enriched in the RS group, while activated dendritic cells and activated mast cells were mainly enriched in the RR group (**Figure 3D**).

### 3.4 Correlation between radiosensitivity signature and treatment sensitivity

**Figure 4:**
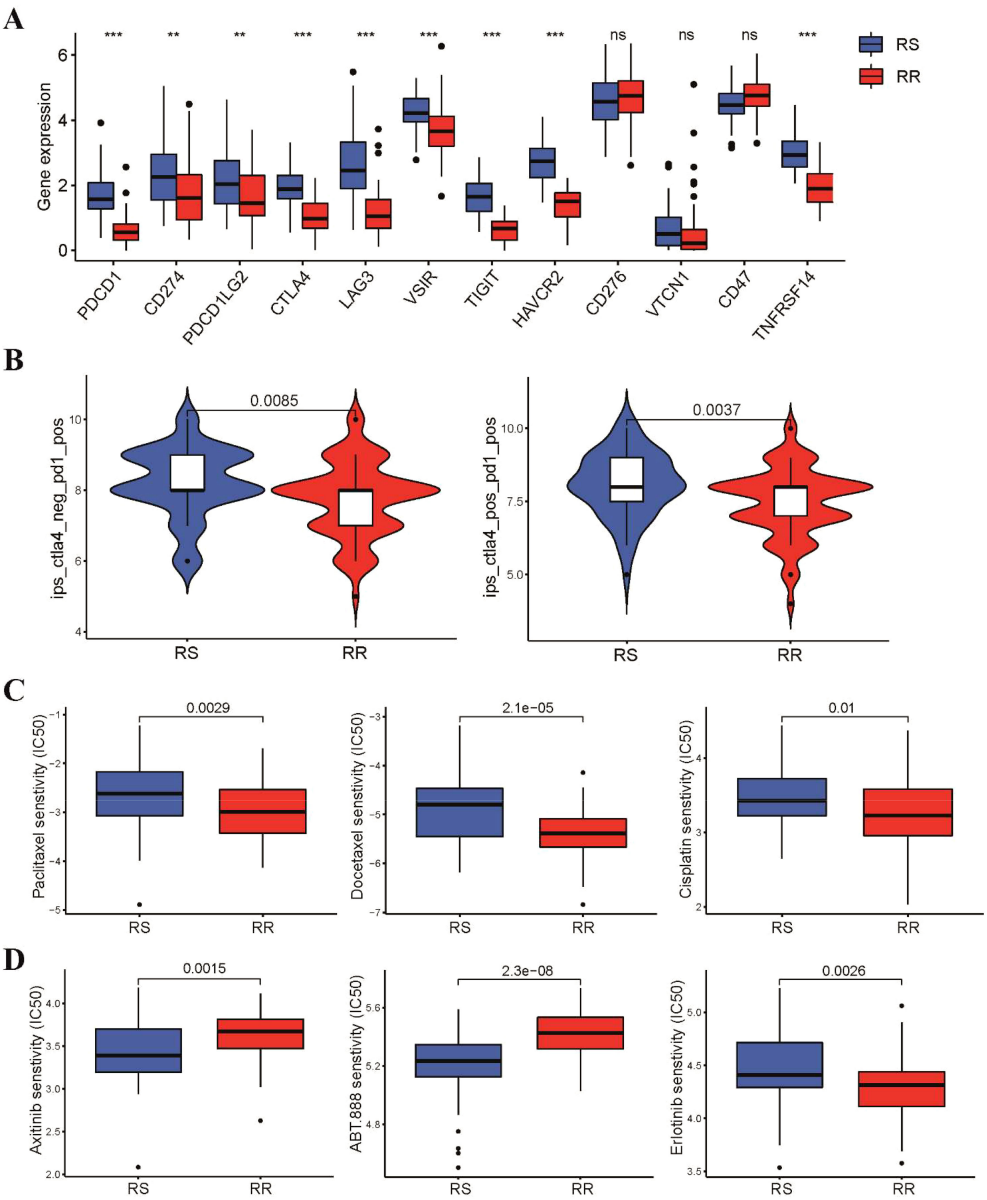
Different sensitivity to immunotherapy, chemotherapy and targeted therapy between two groups. **(A)** Box plots showed the expression level of immune checkpoint genes in the two groups **(B)** Violin diagram showed the Immunophenoscore for CTLA-4 and PD-1 inhibitors between two groups. **(C)** The IC50 values of three first-line chemotherapeutic drugs for TSCC between two groups. **(D)** Difference in IC50 values of targeted therapy drugs for TSCC between two groups. **p < 0.01, ***p < 0.001. ns, not significant.

We further explored the relationship between radiosensitivity signature and patient response to immunotherapy. Our findings revealed that the RS group demonstrated significantly elevated expression levels of immune checkpoint genes (**Figure 4A**), indicating that patients in RS group may be more responsive to immune checkpoint inhibitor. We further employed the Immunophenoscore (IPS) algorithm to validate our previous findings, which implied that patients in the RS group exhibited a superior response to both PD-1 and CTLA-4 inhibitors compared to those in the RR group (**Figure 4B**). In addition, we investigated if these two groups could have varying responses to chemotherapeutics and targeted drugs. To achieve this, we utilized the pRRophetic R package, which leverages the GDSC pharmacogenomic database to predict drug sensitivity. Our results demonstrated that the RS group had higher IC50 values for first-line chemotherapy, including paclitaxel, cisplatin, and docetaxel, compared to the RR group, indicating patients in the RS group may be less responsive to chemotherapy (**Figure 4C**). Additionally, we estimated the IC50 of targeted therapy drugs, which revealed that the IC50 values of VEGFR (Axitinib, Pazopanib, and Sunitinib) and PARP1 (ABT888 and AZD.2281) inhibitors were lower in the RS group. Conversely, the IC50 values of EGFR inhibitors in TSCC patients were significantly higher in the RS group compared to those in the RR group (**Figure 4D** and **Supplementary Figure S1A**).

### 3.5 Validation of the radiosensitivity signature through *in vitro* experiments

**Figure 5:**
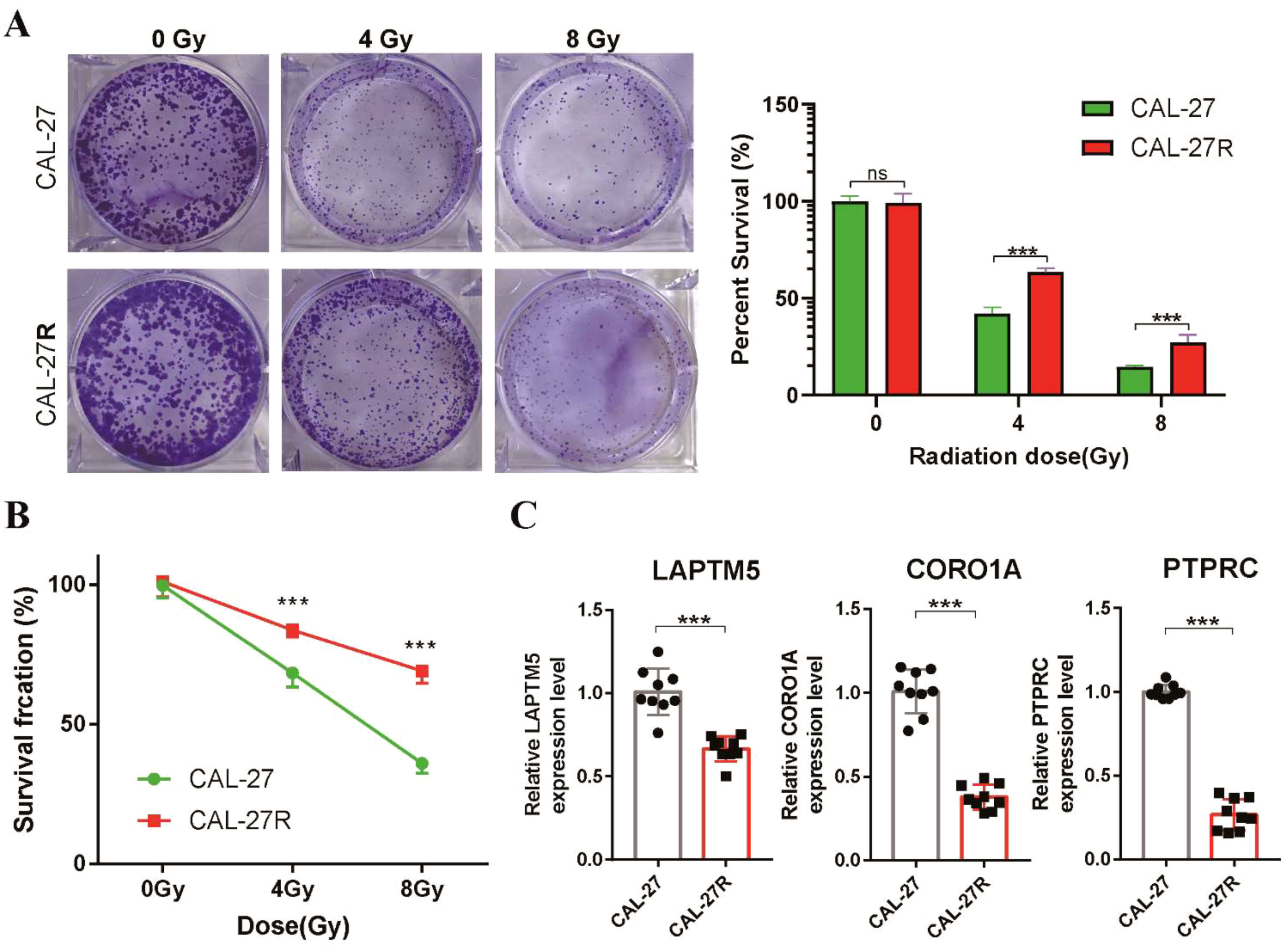
Validation of the radiosensitivity signature through *in vitro* experiments. **(A)** Clonogenic survival of CAL-27 and CAL-27R cells after exposure to 4 and 8 Gy of Radiation. Data presented as bar graph. **(B)** The proliferation ability of CAL-27 and CAL-27R cells after radiation was assessed using the CCK8 assay. **(C)** The expression levels of LAPTM5, CORO1A and PTPRC in CAL-27 and CAL-27R cells were quantified by qRT-PCR. ***p < 0.001. ns, not significant.

To confirm the predictive capacity of the radiosensitivity signature in TSCC, we conducted differential expression analysis between RS and RR groups. Analysis of the two groups identified a total of 108 DEGs, with 19 upregulated and 89 downregulated genes (**Supplementary Figure S2A**). Notably, four genes (LAPTM5, CORO1A, PTPRC and CXCR4) overlapped with the 31 radiosensitivity genes that were increased in the RS group (**Supplementary Figure S2B**). To further investigate these findings, we established a radioresistant TSCC cell line (CAL-27R) from a non-radioresistant TSCC cell line (CAL-27). Clonogenic assays similarly demonstrated a significant reduction in colony formation in CAL-27 cells compared to CAL-27R cells following irradiation. Interestingly, the colonies formed by CAL-27R cells were larger than those derived from CAL-27 cells (**Figure 5A**). CCK-8 assays revealed that CAL-27R cells exhibited increased cell viability compared to CAL-27 cells after exposure to 4 and 8 Gy radiation, confirming the acquisition of radioresistance in CAL-27R (**Figure 5B**). Finally, we verified the expression of the four identified DEGs in CAL-27 and CAL-27R cells. The expression levels of LAPTM5, CORO1A, and PTPRC were significantly higher in the non-resistant CAL-27 cells compared to the resistant CAL-27R cells (**Figure 5C**), which was consistent with our bioinformatics analysis.

### 3.6 Construction of a radiosensitivity-related PI for TSCC patients

**Figure 6:**
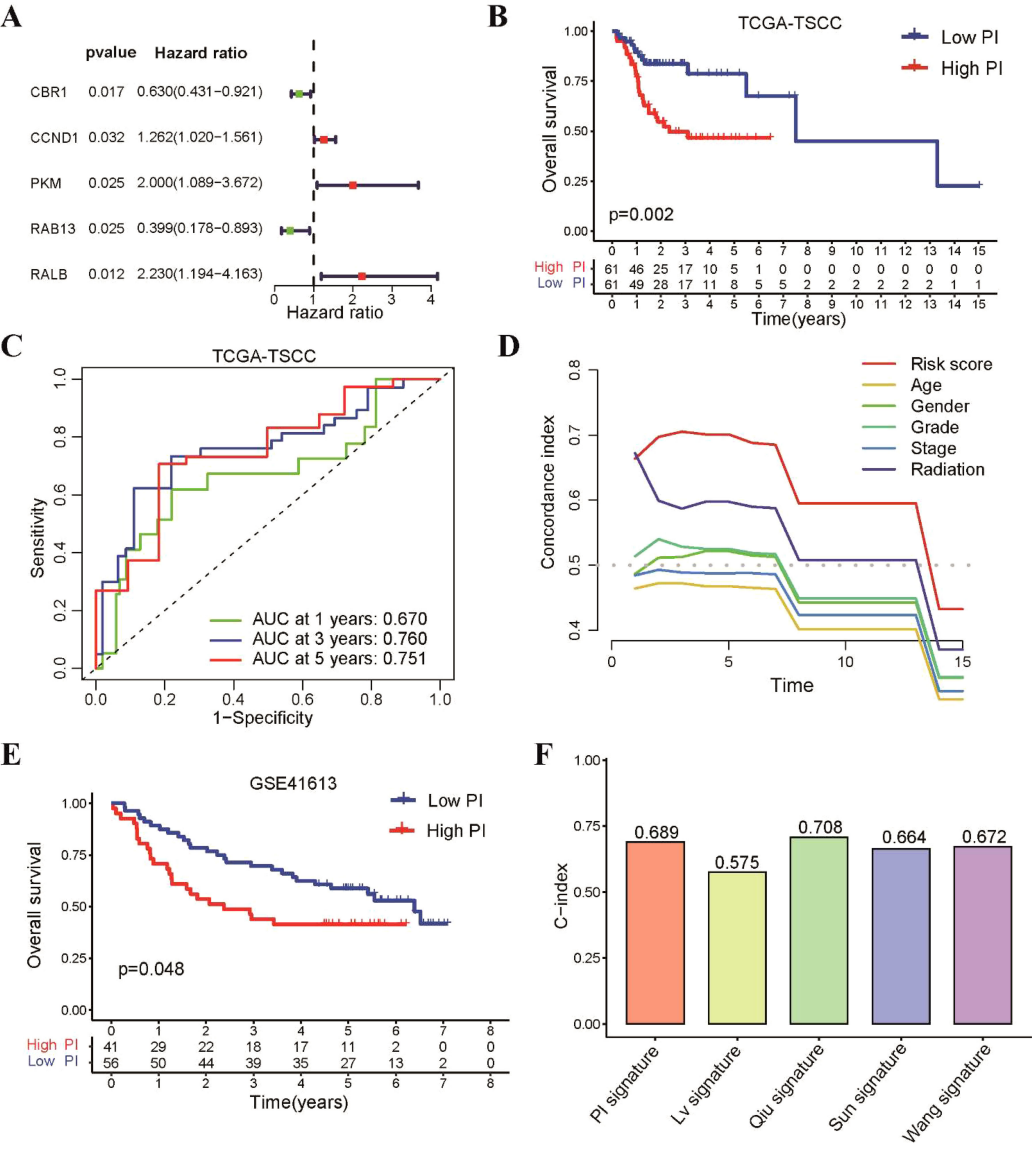
Construction of a prognostic index using 31 radiosensitivity genes for TSCC patients. **(A)** Identification of five prognostic genes based univariate Cox regression analysis. **(B)** Kaplan–Meier curve showing that patients in the low-PI group had a better prognosis than those in the high-PI group in TCGA-TSCC cohort. **(C)** Time-dependent ROC for 1-, 3- and 5-year survival predictions for TSCC patients. **(D)** C-index of the PI and other clinical characteristics. **(E)** Kaplan–Meier curve confirming that patients in the low-PI group had a better prognosis than those in the high-PI group in the GSE41613 cohort. **(F)** The C-index of our PI and four previously prognostic model for TSCC.

Since the 31 radiosensitivity genes were derived from the NCI-60 cancer cell line panel which is not specific to tongue cancer, we constructed a radiosensitivity-related prognostic index (PI) for TSCC patients using 31 radiosensitivity genes (11, 16). The univariate Cox regression analysis identified 5 genes associated with the prognosis of TSCC (**Figure 6A**). Subsequently, The LASSO-Cox regression analysis identified that 4 genes were used to build the prognostic model, which were CBR1, CCND1, RAB13 and RALB (**Supplementary Figure S3A**). The prognostic index formula was as follows: PI = (-0.4096 × mRNA level of CBR1) + (0.2129 × mRNA level of CCND1) + (-0.7336 × mRNA level of RAB13) + (0.7147 × mRNA level of RALB) (**Supplementary Figure S3B**). Patients were classified into the high-PI group and low-PI group based on the median value of the PI score (**Supplementary Figure S3C**). The statistical analysis revealed a significant difference in the expression levels of these four genes between the high-PI and low-PI groups (**Supplementary Figure S4A**). The Kaplan-Meier survival curve demonstrated that patients of low-PI group exhibited better OS than those in the high-PI group (**Figure 6B**). Receiver operating characteristic (ROC) curves illustrated that the AUC values for 1-, 3- and 5-year were 0.67, 0.76 and 0.751, respectively (**Figure 6C**). The C-index of the PI was higher than other clinical characteristics, including gender, age, grade, stage and radiotherapy, suggesting that the model has better discriminative ability than other clinical characteristics (**Figure 6D**).

To assess the predictive accuracy of our prognostic model, the GSE41613 dataset was utilized as an external validation cohort. TSCC patients from this dataset were divided into low-PI and high-PI groups according to the PI formula. These findings aligned with those observed in the TCGA-TSCC cohort, there was a consistent trend and statistically significant difference in the expression levels of these hub genes between the high-PI and low-PI groups in GSE41613 dataset (**Supplementary Figure S4B**). Notably, the low-PI group showed a notably better survival rate compared to the high-PI group (**Figure 6E**). To further assess the model’s validity, we compared its performance to four previously published prognostic signatures for TSCC. Our results revealed that our PI had a higher C-index and AUC value than the other signatures (**Figure 6F** and **Supplementary Figure S5**).

### 3.7 Development of a nomogram to predict the prognosis of TSCC patients

**Figure 7:**
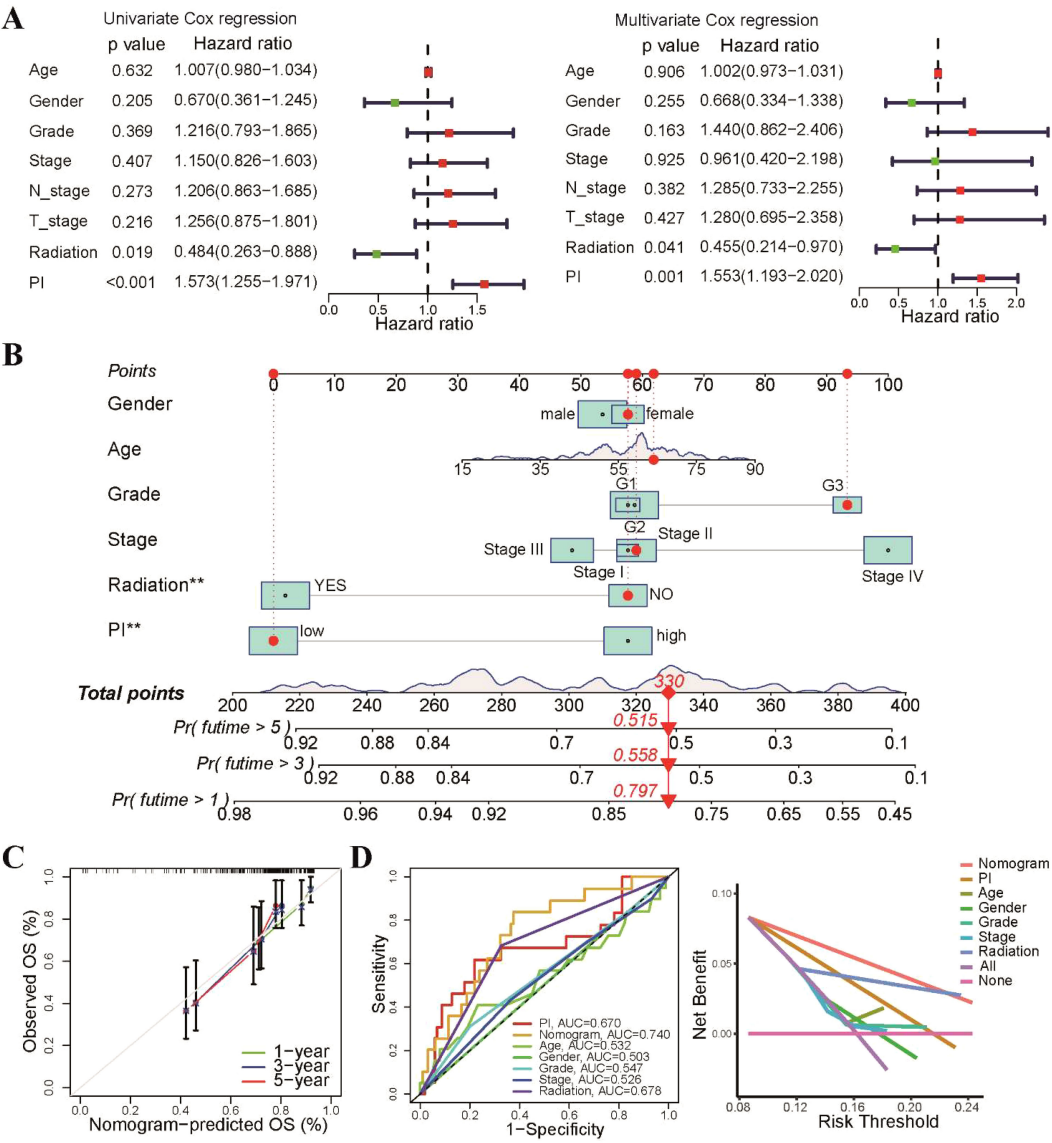
Development of a predictive nomogram based on radiosensitivity PI. **(A)** Univariate and multivariate Cox regression analyses demonstrated that the PI was an independent prognostic factor for patients with TSCC. **(B)** The prognostic nomogram was based on the PI and common clinical characteristics. **(C)** The calibration plot displayed that the nomogram-predicted probabilities were closely aligned with actual survival rates. **(D)** The ROC and DCA curves showing the performance of the nomogram and different clinical characteristics. **p < 0.01.

Consequently, we evaluated the independent prognostic impact of our radiosensitivity-related prognostic model on OS using both univariate and multivariate Cox regression analyses. The results demonstrated that the PI was an independent factor for TSCC patient (**Figure 7A**). We developed a nomogram to enhance the clinical applicability of the PI prognostic model by incorporating PI and common clinical characteristics, such as gender, age, grade, stage, and radiotherapy (**Figure 7B**). The calibration curves exhibited excellent agreement between the predicted probabilities from the nomogram and the survival rates (**Figure 7C**). The ROC and decision curve analysis (DCA) curves also indicated that the nomogram had a relatively high discriminative ability in predicting the prognosis of TSCC patients (**Figure 7D**). Taken together, these findings suggest that the nomogram could serve as a practical tool and offer valuable guidance for TSCC patients in clinical practice.

### 3.8 Immune profile differences in high-PI and low-PI groups

**Figure 8:**
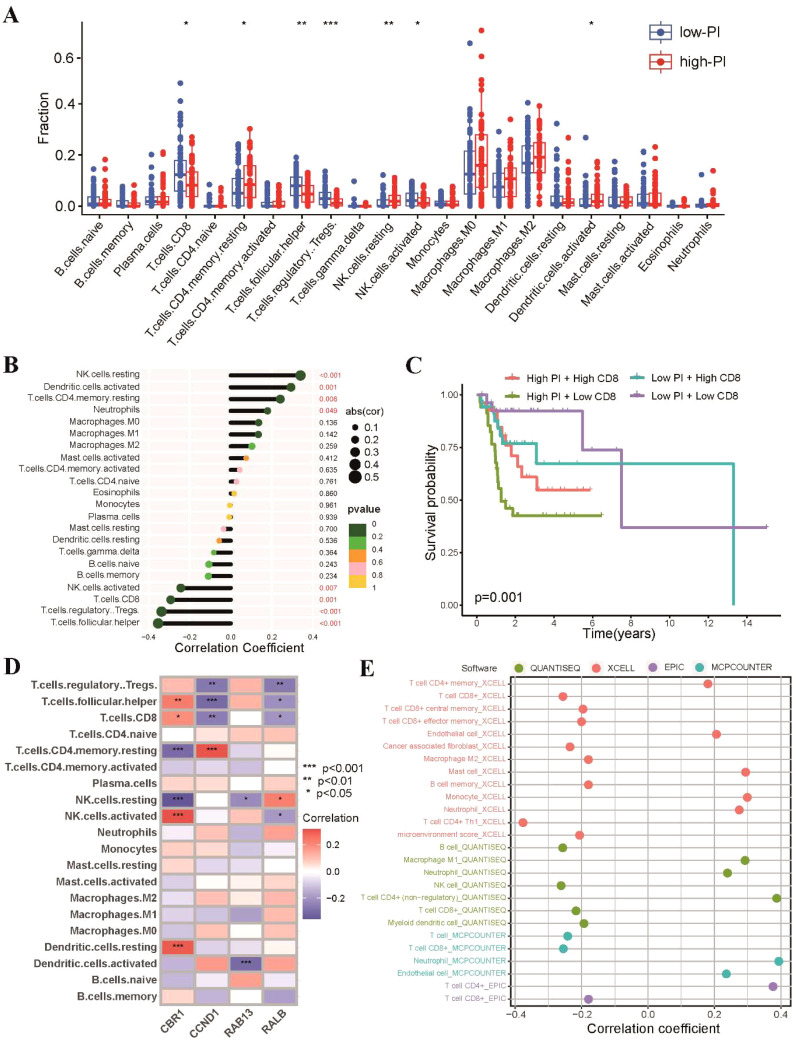
Landscape of tumor immune profile between high-PI and low-PI groups. **(A)** Comparisons of the proportions of 22 immune-infiltrating cells between the high-PI and low-PI groups. **(B)** Correlation analysis between immune-infiltrating cells and PI. **(C)** Kaplan-Meier survival curves in different groups according to the abundances of CD8 T cells. **(D)** Associations between the expression level of the four genes of PI and 22 tumor-infiltrating immune cells. **(E)** Correlations between PI and tumor-infiltrating immune cells of TSCC analyzed by QUANTISEQ, XCELL, EPIC and MCPCOUNTER. * p<0.05, ** p<0.01, *** p<0.001.

As radiosensitivity is associated with immune status of tumors, we investigated the tumor immune microenvironment of the high-PI and low-PI groups. Firstly, we utilized the CIBERSORT algorithm to estimate 22 tumor-infiltrating immune cells of TSCC. Consistent with the RS group, the low-PI group exhibited higher proportions of CD8 T cells, regulatory T cells, and Tfh cells, while the high-PI group showed elevated levels of resting memory CD4 T cells, activated dendritic cells, and resting NK cells (**Figure 8A**). A negative correlation was observed between PI and CD8 T cells, regulatory T cells, and Tfh cells, while resting memory CD4 T cells, activated dendritic cells, and resting NK cells showed a positive association with PI (**Figure 8B**). We found that patients with higher CD8 T cell abundances in the low-PI group had better survival rates than those with lower CD8 T cell abundances in the high-PI group (**Figure 8C**). The associations between the expression level of the 4 genes of PI and 22 tumor-infiltrating immune cells in TSCC was shown in **Figure 8D**. Moreover, we further estimated immune cell infiltrations of TSCC through QUANTISEQ, XCELL, EPIC and MCPCOUNTER algorithms to systematically explored the relationship between PI and tumor-infiltrating immune cells. The results exhibited that radiosensitivity-related PI was negatively correlated with abundance of CD8 T cells in all five algorithms (**Figure 8E**).

## 4 Discussion

Radiation therapy is a crucial treatment modality for patients with TSCC (27). However, patients demonstrate significant variability in their sensitivity to radiotherapy, with some responding more favorably to radiation than others. Consequently, it is imperative to identify predictive markers of radiosensitivity to optimize radiation therapy. Precision medicine has emerged as a powerful approach to personalize treatment strategies, and identifying potential biomarkers is a key focus of this field (28). This study aimed to evaluate the predictive power of a 31-gene signature, proposed to reflect radiosensitivity, using the TCGA-TSCC dataset. Our findings indicate that this gene signature may have potential as a predictive marker for radiotherapy response in TSCC patients. Additionally, we developed the radiosensitivity-related prognostic index and nomogram that could provide valuable guidance for TSCC patients in clinical practice. These tools could serve as practical aids to optimize treatment strategies and improve clinical outcomes for TSCC patients.

Radiosensitivity is a term whose definition varies depending on the context in which it is used. In clinical settings, radiosensitivity is defined based on two criteria: firstly, the survival rate of the RS group should not be better than that of the RR group when neither group receives radiotherapy. Secondly, when both groups receive radiotherapy, the RS group should experience significantly more survival benefits than the RR group (29). The 31-gene signature has been validated for predicting radiosensitivity in glioblastoma, breast cancer, and low-grade gliomas (12–14). In this study, we tested the 31-gene signature as a predictive biomarker for predicting radiosensitivity in patients with TSCC receiving radiotherapy, which to our knowledge, is the first such study. In recent years, several studies have identified radiosensitive biomarkers for predicting radiotherapy outcomes in HNSCC patients. For example, Liu et al. developed a 12-gene signature using multiple omics data, which showed superior predictive power for radiosensitivity (30). Similarly, Ma et al. developed a methylation-based signature consisting of four genes, which proved to be a valuable predictor of survival in HNSCC patients receiving radiotherapy (31). However, selecting TSCC patients who will benefit most from radiotherapy remains a challenge due to the lack of reliable radiosensitivity biomarkers. Our study employed consensus clustering to classify patients into RS and RR groups. We found that the RS group had better OS and PFI rates than the RR group when treated with radiotherapy, but not when untreated. Our findings suggest that the radiosensitivity gene signature could be a valuable tool for predicting the response to radiotherapy in TSCC patients.

Radiation is known to activate the immunologic response within the tumor microenvironment, which dynamically changes in response to radiotherapy (32). Thus, comprehending the impact of immune microenvironment function on the efficacy of radiotherapy is crucial for optimizing treatment strategies. Previous research on radiosensitivity has focused primarily on tumor cells, neglecting the role of stromal and immune cells within the tumor microenvironment (33, 34). Recent studies have revealed the significant influence of the immune system on cancer patients’ response to treatment and long-term prognosis. For instance, Yan et al. developed an immune-related radiosensitivity gene signature to predict the survival of lower grade glioma patients who received radiotherapy (35), while Mathias Fiedler et al. found that T-cell activation is associated with radio response and favorable survival in advanced head and neck cancer treated with definitive radiotherapy or chemoradiation (36). Consistent with these findings, our results demonstrate that the RS group had significantly enriched immune-related hallmark pathways and high T-cell infiltration, particularly CD8 T cells and Tfh cells, which were prognostic factors for better outcomes. Among immune cells, CD8+ T cells, also known as cytotoxic T lymphocytes, play a critical role in targeting and eliminating cancer cells. One explanation for this is that radiation dramatically increases cytotoxic T-cell infiltration, which not only indicates a better response but also results in better tumor shrinkage (37).

Interestingly, our study found an association between high Treg cells and the RS group. The immunosuppressive activity of Tregs within the tumor microenvironment is often associated with unfavorable outcomes in most solid tumors (38). However, the contradictory finding of high Treg cells in the RS group may indicate a robust T-cell response within the tumor, suggesting a more effective antitumor immunity. While Tregs have immunosuppressive effects, the presence of a substantial population of CD8+ T cells could have a dominant influence. Moreover, as a “hot” immune-inflamed microenvironment is typically associated with high CD8 T cell counts, predicting benefit from the PD-1/CTLA-4 blockade (33). Our study observed an enrichment of TCIA in TSCC patients of the RS group, but not in the RR group. These findings offer a potential explanation for the limited efficacy of combined radiotherapy and immunotherapy in certain patients, particularly those in the RR group. Effective checkpoint inhibitor therapy requires sufficient T-cell infiltration within the tumor, a factor that may be lacking in these patients. In contrast, patients in the RR group showed higher sensitivity to first-line chemotherapeutic drugs, including paclitaxel, cisplatin, and docetaxel. Accordingly, a combination strategy involving chemotherapy with radiotherapy has been proposed as a means to overcome radio-resistance in these patients.

Lastly, we have developed a radiosensitivity-related prognostic index by utilizing the aforementioned radiosensitivity genes. Our signature is composed of five radiosensitivity genes, including CBR1, CCND1, RAB13, PKM, and RALB, which have been extensively studied and found to play crucial roles in regulating cellular responses to radiation. For instance, CBR1 has been shown to be involved in the production of reactive oxygen species (ROS) that can induce DNA damage (39), while CCND1 has been found to control the cell cycle and promote DNA repair (40). Similarly, RAB13 has been implicated in the regulation of DNA damage response pathways (41), and PKM has been shown to modulate cellular metabolism and DNA damage repair (42). RALB has been found to play a role in the regulation of cellular migration and invasion, which can influence the response to radiation therapy (43).

The present study was not without its limitations. Firstly, the retrospective nature of the database used, The Cancer Genome Atlas (TCGA), may have introduced potential bias due to variations in follow-up information. Therefore, a prospective study involving a homogenous cohort of TSCC patients is needed to validate our findings. Secondly, the relatively small sample size may have limited the statistical power of our analyses and restricted the generalizability of our findings to a broader population, underscoring the need for caution in interpreting the results. Therefore, future research involving a larger cohort is essential to validate our findings and confirm their applicability across diverse patient populations. Thirdly, the estimation of immune cell infiltration in the tumor microenvironment, rather than its direct measurement, is a limitation of the study. Lastly, the selection of the 31 radiosensitivity genes from the NCI-60 cancer cell line panel, which does not include tongue cancer cell lines, may raise concerns regarding the relevance and specificity of these genes in dividing TSCC patients. However, it is important to note that epithelial malignancies possess certain commonalities in terms of radiation response. Therefore, the significance of these genes lies in their ability to provide insights into the broader mechanisms of radiation response in epithelial tumors. Nevertheless, additional investigations specifically targeting TSCC patients are imperative in order to validate the relevance and clinical applicability of these gene signatures.

## 5 Conclusion

In summary, this study aimed to validate a previously identified “31-gene signature” linked to radiosensitivity in TSCC and its potential in predicting radiotherapy response and prognosis. Patients in the RS group demonstrated improved overall survival and progression-free survival rates compared to those in the RR group when treated with radiotherapy. Moreover, the RS group showed significant enrichment in most immune-related hallmark pathways, suggesting potential benefits from immune checkpoint inhibitors. However, the RS group demonstrated lower sensitivity to first-line chemotherapy. The study also developed and validated a radiosensitivity-related prognostic index utilizing four radiosensitivity-related genes associated with TSCC prognosis. Overall, the study provides valuable insights into the molecular pathways associated with radiosensitivity in TSCC and offers clinicians a practical tool to predict patient radiotherapy effectiveness and prognosis.

## Data Availability

The original contributions presented in the study are included in the article/**Supplementary Material**. Further inquiries can be directed to the corresponding authors.
