# Supplementary material for: Predicting radiotherapy efficacy and prognosis in tongue squamous cell carcinoma through an in-depth analysis of a radiosensitivity gene signature
Source: Front Oncol. 2024 Aug 26;14:1334747. doi: 10.3389/fonc.2024.1334747 (PMC11381225; doi:10.3389/fonc.2024.1334747)
Supplement: Supplementary file 1 [file DataSheet1.docx]

**
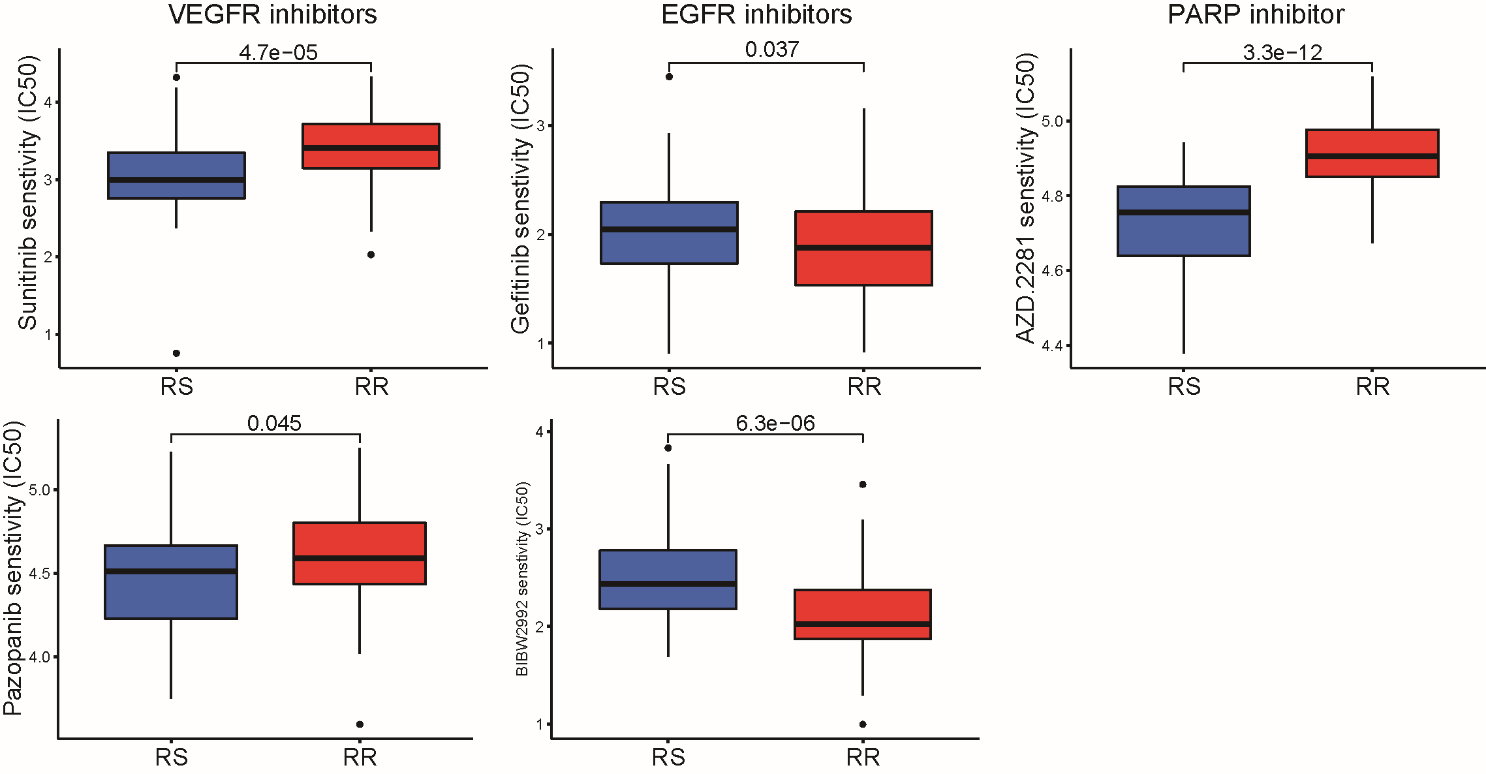
**

**Figure S1**: Different sensitivity to targeted therapy drugs between two groups. Difference in IC50 values of VEGFR (Pazopanib and Sunitinib), PARP1 (AZD.2281) and EGFR (Gefitinib and Afatinib) inhibitors for TSCC between two groups.


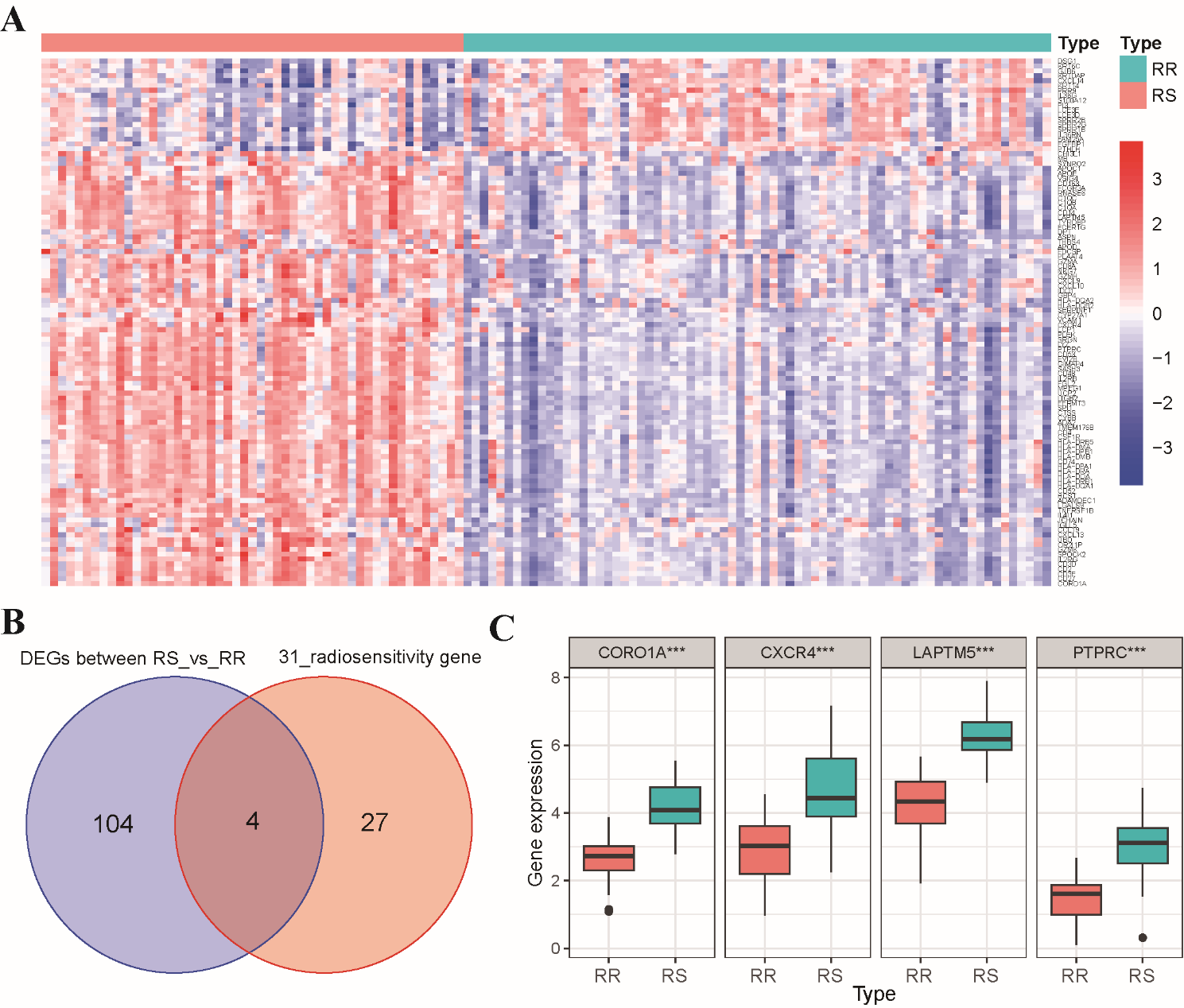


**Figure S2**: **(A)** Heatmap displaying the expression of DEGs between RS and RR groups. **(B)** Venn diagram demonstrating the overlap of four genes between the 108 DEGs and 31 radiosensitivity genes. **(C)** The expression levels of the four overlapping genes in the TCGA dataset for the RS and RR groups.


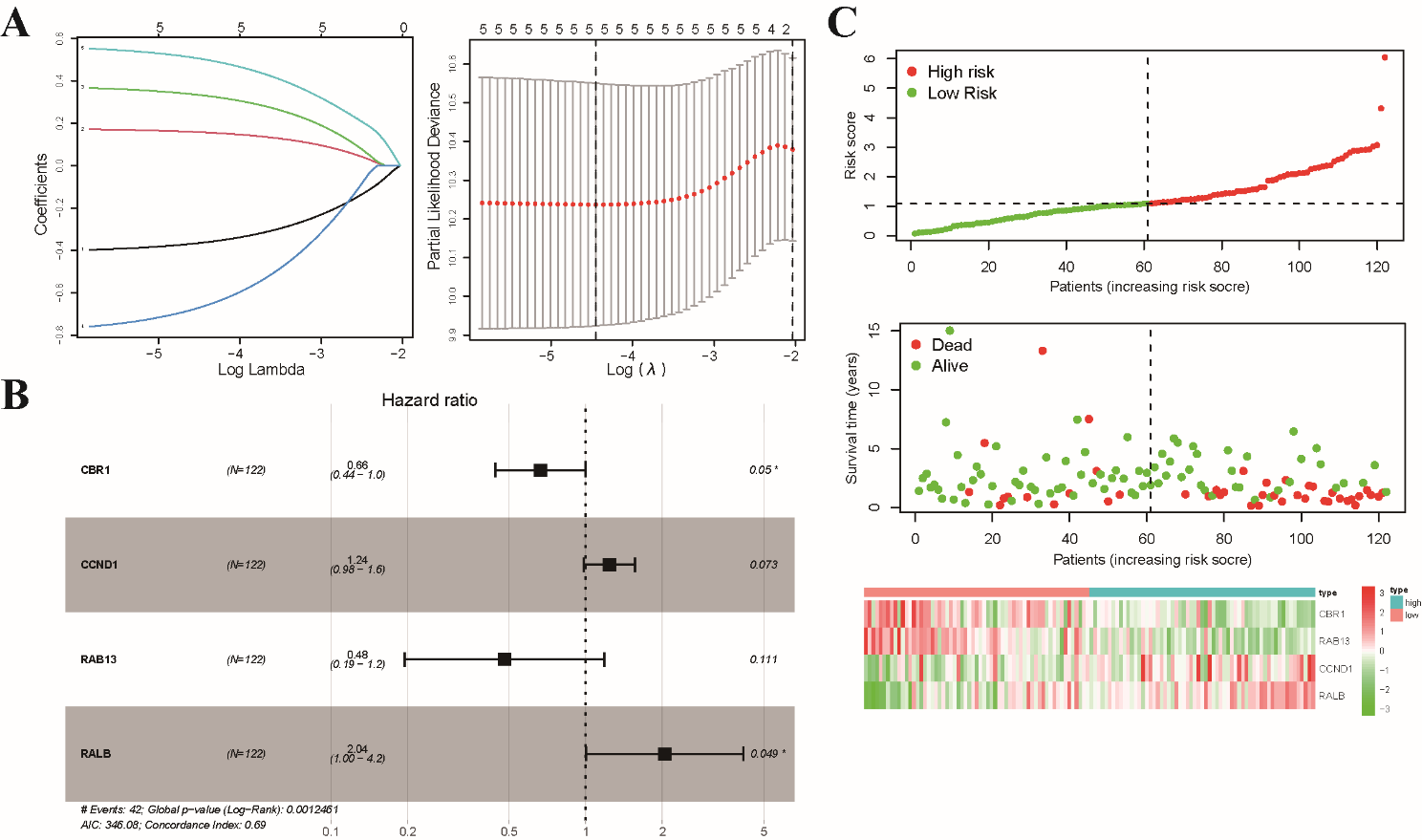


**Figure S3**: **(A)** The LASSO regression analysis was utilized to select most useful prognostic genes. **(B)** Forest plot of the multivariate Cox regression analysis in the TCGA-TSCC cohort. **(C)** The distribution of risk scores, survival status and genes expression panel in the TCGA-TSCC cohort.


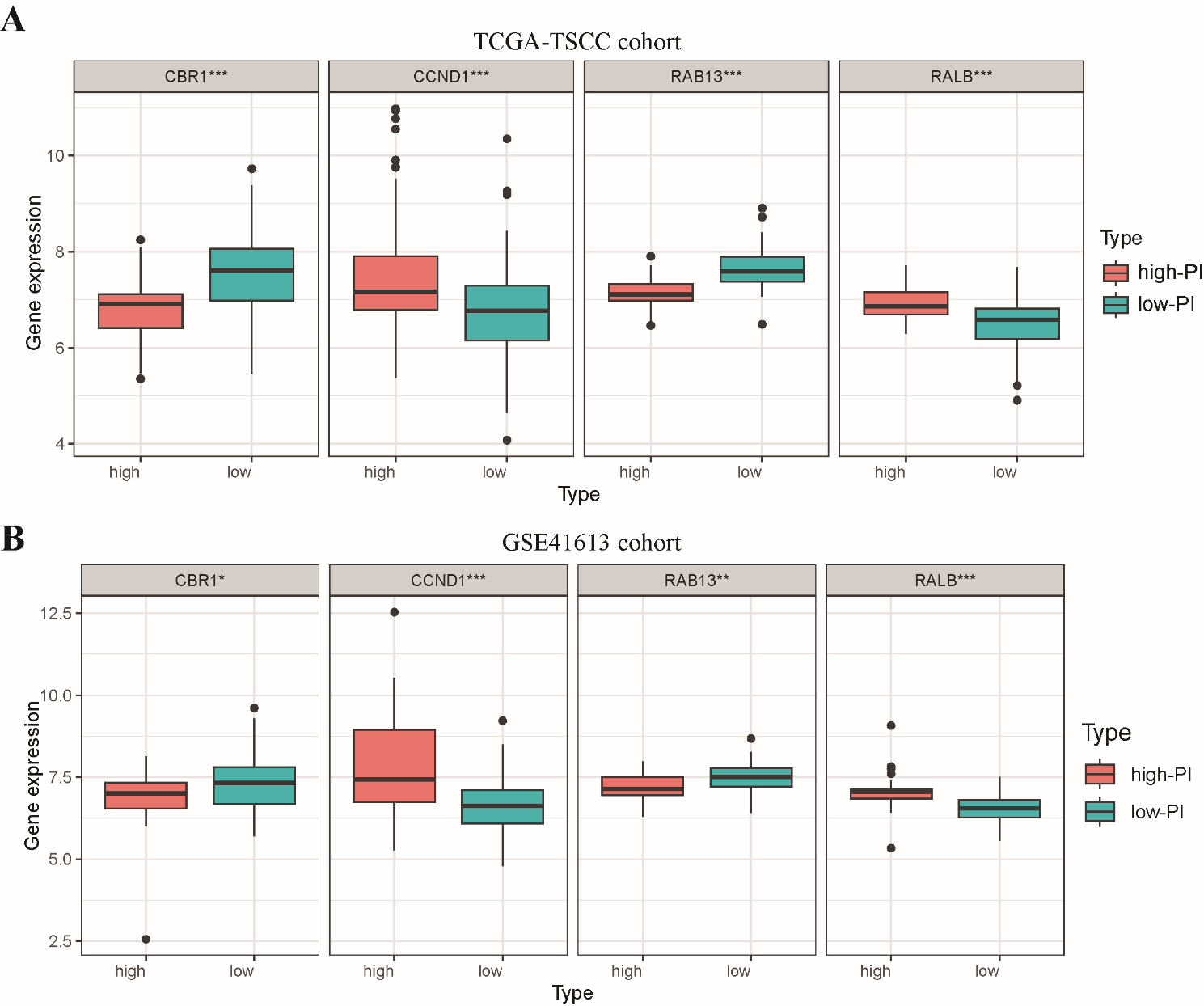


**Figure S4**: **The comparison of these five genes between the high-PI and low-PI groups.** **(A)** There was a significant difference in the expression levels of these five genes between the high-PI and low-PI groups in TCGA-TSCC cohort. **(B)** In the GSE41613 dataset, a statistical difference was observed when comparing these hub genes between the high-PI and low-PI groups, except for the RAB13 gene.


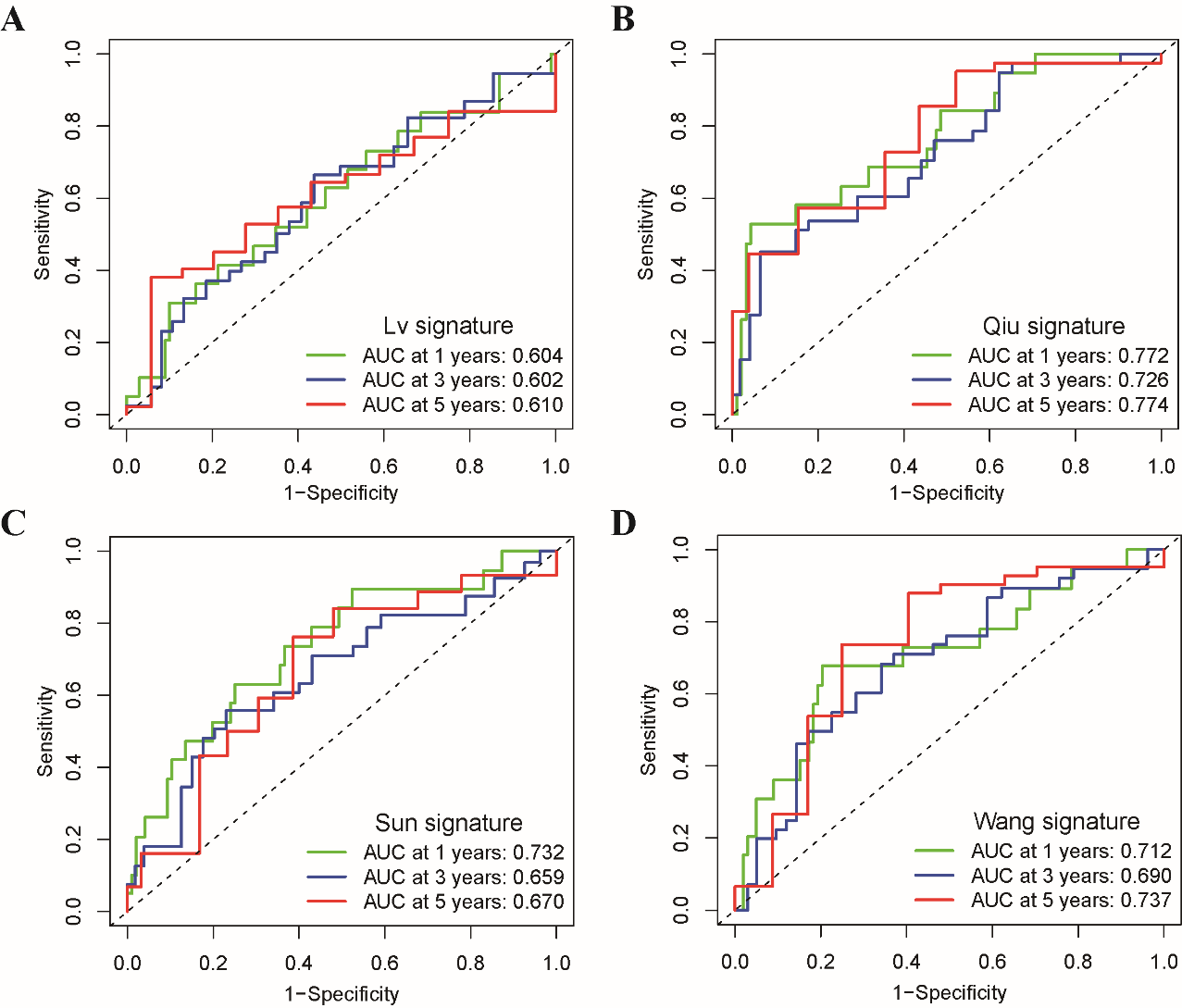


**Figure S5**: The time-dependent ROC for 1-, 3- and 5-year survival predictions for TSCC patients in LV signature **(A)**, Qiu signature **(B)**, Sun signature **(C)** and Wang signature **(D)**.

**TABLE S1 |** Sequences of the primer used for qRT-PCR

| mRNA | Forward primer | Reverse primer |
| --- | --- | --- |
| LAPTM5 | CCTGAGCCTACTGATCGGC | CAGGCACAGGAGATAGTCCA |
| CORO1A | CTACTTGGGAGGGGTCACG | TTTGCTGGAGCGAACCAC |
| PTPRC | ATGATGGCAGGGTCCTTCATGT | AGCTTCCTCAGCTAATGATTGGACA |
| GAPDH | CCAACTGCCAGACTACCAC | GGACCAGGCTGTTCCAAGA |
